# Psychosocial Profile and Reproductive Decisions of Women Undergoing Pregnancy Termination for Medical Reasons—A Cross-Sectional Study

**DOI:** 10.3390/ijerph16183413

**Published:** 2019-09-14

**Authors:** Kornelia Zaręba, Valentina Lucia La Rosa, Michał Ciebiera, Marta Makara-Studzińska, Jacek Gierus, Grzegorz Jakiel

**Affiliations:** 1 I Department of Obstetrics and Gynecology, Center of Postgraduate Medical Education, 01-004 Warsaw, Poland; 2 Unit of Psychodiagnostics and Clinical Psychology, University of Catania, 95124 Catania, Italy; 3 II Department of Obstetrics and Gynecology, Center of Postgraduate Medical Education, 01-809 Warsaw, Poland; 4 Faculty of Clinical Health Psychology Jagiellonian University Medical College, 31-126 Krakow, Poland; 5 Faculty of Psychology, University of Economics and Human Sciences, 01-043 Warsaw, Poland

**Keywords:** pregnancy termination, abortion, fetal defects, psychosocial profile, decision determinant, choice

## Abstract

Background: The study aims to define the profile of women who decide to exercise their right to terminate a pregnancy and their future reproductive plans. Methods: Patients found eligible for termination for medical reasons between 2014 and 2016 were asked to complete an anonymous survey consisting of sixty questions examining the determinants of the decision to terminate a pregnancy. In total, 150 completed surveys were returned (62.5%). Results: Environmental factors, such as age, education, place of residence, marital status and financial status did not affect the decision-making process. The majority of the respondents were females under 35 years of age (71.3%). In most cases, the pregnancies had been planned and long-awaited (62.7%). The study also indicated that 22.6% of the patients who had been against abortion changed their mind when they encountered problems themselves. In addition, 20% of them changed their views on the acceptability of abortion. Termination had an impact on the participants future reproductive plans. Eighteen percent of the patients said they were definitely not planning more pregnancies. The majority (84.09%) of these women said that the reason was the traumatic experiences related to their pregnancy. Conclusions: The personal experience of a pregnancy termination procedure changed women’s opinions about pregnancy termination and modified further reproductive plans.

## 1. Introduction

Around 120–150 terminations per year are performed in the I Department of Obstetrics and Gynecology, Center of Postgraduate Medical Education, Warsaw, Poland. Almost all of these procedures in Poland are performed due to irreversible fetal defects that lead to severe fetal damage or death of the child after birth [1].

Compared to other European countries, Polish law is one of the most restrictive for abortion. Similar to other ex-Soviet Union states, legal abortion has been widely available since 1956. However, an act issued in 1993 set out significantly stricter indications for termination of pregnancy, restricting them to strict medical reasons and sexual offences. The procedure can be performed until the 22nd week of pregnancy [2,3]. Legal abortion may be performed in one of the following instances:

1. The pregnancy poses a direct threat to maternal health or life (before the fetus is able to survive outside the uterus), confirmed by a doctor other than the one performing the procedure.
2. Prenatal screening or other medical evidence indicates a high probability of severe and irreversible fetal anomaly or incurable life-threatening disease (before the fetus is able to survive outside the uterus), confirmed by a doctor other than the one performing the procedure.
3. There is a reason to believe that the pregnancy (up to 12 weeks of gestation) is the result of an unlawful act, confirmed by a prosecutor.

In practice, few terminations are performed in Poland due to the above maternal indications. Sociodemographic factors such as age, other children, ethnic origin, religion, educational background, marital status, profession, views on abortion, and relations with partners have various impacts on the decision to undergo abortion, depending on the research methodology, country and reasons for making the decision to terminate the pregnancy [4,5,6,7]. Moreover, numerous external factors influence the process of making a decision about termination of a pregnancy: legislation, the healthcare system, scope of medical insurance, access to healthcare services, activity of support groups, social position and access to medical information [8,9].

According to Polish public opinion, pregnancy termination for medical reasons is not well accepted, and this view is also shared by some members of the medical community [2,10]. This attitude differentiates Poland from many other countries and creates an interesting situation from a research perspective. Patients who decide to terminate a pregnancy are aware of their own autonomy and exercise their rights within this context.

In light of these considerations, the main aim of this study was to determine a profile of women who decide to exercise their right to terminate a pregnancy. Moreover, we aimed to determine whether financial and sociodemographic factors had an influence or whether political views had an influence on making the decision to terminate a pregnancy.

## 2. Materials and Methods 

At the initial stage, a consent to perform the study was obtained from the Ethical Board at the Center of Postgraduate Medical Education, Warsaw (ethical approval code: 78/PB/2014). A board composed of four members (Head of the Department and three specialised physicians) verified the eligibility of patients requesting termination of pregnancy for medical reasons at the I Department of Obstetrics and Gynecology, the Center of Postgraduate Medical Education, Warsaw, Poland. After the verification of medical reason(s) for termination, in accordance with Article 4a, Item 2 of the Act on Family Planning, Human Embryo Protection and Conditions of Permissibility of Abortion as of 7 January 1993 [1], all patients admitted to the hospital between 1 June 2014 and 31 May 2016 were asked to complete an anonymous survey consisting of sixty questions. Each patient enrolled in the study, signed informed consent for all the procedures and to allow data collection and analysis for research purposes. The study was not advertised, and no remuneration was offered to the patients to enter or continue the study. An independent data safety and monitoring committee evaluated the results. The design, analysis, interpretation of data, drafting and revisions conform to the Declaration of Helsinki, the Committee on Publication Ethics (COPE) guidelines and the Strengthening the Reporting of Observational Studies in Epidemiology (STROBE) statements.

### 2.1. Study Group Inclusion Criteria

- Patients asking for termination of pregnancy presenting at our department.
- Presence of indications for termination in accordance with the Act on Family Planning, Human Embryo Protection and Conditions of Permissibility of Abortion as of 7 January 1993 [1].
- Consent to participate in the study.

### 2.2. Survey

The questions used for the study and the characteristics of the group were part of a larger survey created to determine the psychosocial profiles of women who underwent pregnancy terminations due to medical indications in our hospital [11,12]. Due to the large size of the survey and the wide scope of the issue, we have presented the results in independent articles that describe different aspects of the study. After the verification of the medical grounds for termination in accordance with the law, all the patients admitted to the hospital were asked to complete an anonymous survey consisting of sixty questions. The patients were recruited prospectively. A physician or a midwife asked the patient to complete the survey. To eliminate the medical staff’s impact on the responses, the patients completed the survey in private (during their hospital stay). The termination procedure was performed afterwards. Surveys were returned at the time of discharge from the hospital. The survey was created especially for the purposes of the study in cooperation with a clinical psychologist participating in the study. The survey consisted of six sections: general information, general medical interview, pregnancy-based medical interview, religion, outlook on life, support and moral dilemmas. Some answers were provided on a five-point Likert scale with a indicating strong agreement and e indicating strong disagreement. It took the patients around thirty minutes to complete the questionnaire. The physician provided assistance in case of any doubts. In total, one hundred and fifty surveys were collected. The total return rate was 62.5%.

### 2.3. Statistical Analysis

Prior to conducting the final analyses, preliminary analyses were performed, which included descriptive statistics with frequency expressed as numbers and percentages, means, standard deviations. Categorical variables were expressed as frequencies and percentages, and distributions were represented with graphs and tables. Spearman’s rank correlation coefficient was also used in the majority of cases in order to examine the strength of the patients’ views on the Likert scale. The criterion of statistical inference was set at the level of significance of *p* < 0.05. No assumption of inter-group differences was stated (the group included only those patients who decided to terminate a pregnancy), so no test of differences was used. Chi-square-based normality tests (Shapiro-Wilk, Kolmogorov-Smirnov) were conducted and no distributions were normal. The analyses were conducted with the STATISTICA 13.1 (StatSoft, Krakow, Poland) statistical package. 

## 3. Results

### 3.1. Social Characteristics

The study group consisted of 150 patients aged 18–45. The majority of the patients (over 50%) were aged 25–35. Women with a low population risk for genetic defects, i.e., under 35 years old, constituted 71.3% of the respondents. Women with an increased risk for genetic defects represented only 28.7% of all respondents. Almost all patients resided in a city (95.3%) (Table 1) [12].

More than half of the patients (52.7%) had higher education degrees. Only three patients reported only having completed primary education. The rest of the women had secondary or incomplete higher education qualifications (Table 1). The majority of the patients’ parents had secondary education qualifications, with a small percentage having completed higher or incomplete higher education [12]. No impact of the parents’ educational background on the decision was noticed. The highest percentage of the respondents worked in healthcare, education, banking and finance, i.e., professional groups with a higher educational background. Only 10.7% of the respondents reported financial difficulties (Table 1). The majority of the patients (72%) stated that their standard of living was that of the middle class. The majority of patients said they were in a committed relationship (98.7%), while 79.3% were married. Therefore, nearly 100% of the respondents had a stable relationship status [12].

Political views did not influence the decision. The majority of patients, i.e., 21.3%, were liberal right-wing supporters. However, it should be noted that 35.3% of the respondents did not answer the question at all, while 20.7% of the respondents chose “other”, with many of them indicating that they were not interested in politics at all (over 55% of the respondents were not politically-oriented).

### 3.2. Medical Characteristics

In our study group, termination of pregnancy was performed mainly due to genetic defects (50.7%), followed by severe malformation syndromes (13.3%) and defects of the central nervous system (15.3%). Out of the genetic defects, trisomies were the most common (including Trisomy 21 (42%), Trisomy 18 (23%) and Trisomy 13 (8%)), followed by other triploidies (15%).

Only 16.7% of the patients reported a chronic illness. The most common diseases were asthma (2.7%) and allergies (1.3%). The majority (86.7%) of the respondents indicated that they visited a gynecologist for check-ups at least once a year. Similarly, 72.7% of the respondents had pap smears performed every year, and 95% of the respondents had a pap smear every few years, according to the recommended intervals. This confirms the hypothesis of the awareness of the need for medical prophylaxis in the study group. The vast majority of patients reported to every scheduled visit during their current pregnancy (97.3%). Patients with no children constituted 42.7% of the respondents. Among multigravida patients, the majority had one child (39.3%). The group of respondents also included a patient who had given birth eight times. One-third (31.3%) of the patients reported a history of miscarriage, with the highest number of miscarriages being four. Only one patient reported having had an ectopic pregnancy. A significant obstetric history (understood as at least three past miscarriages) was given by four patients, which constituted 2.7% of the study group.

**Figure 1:**
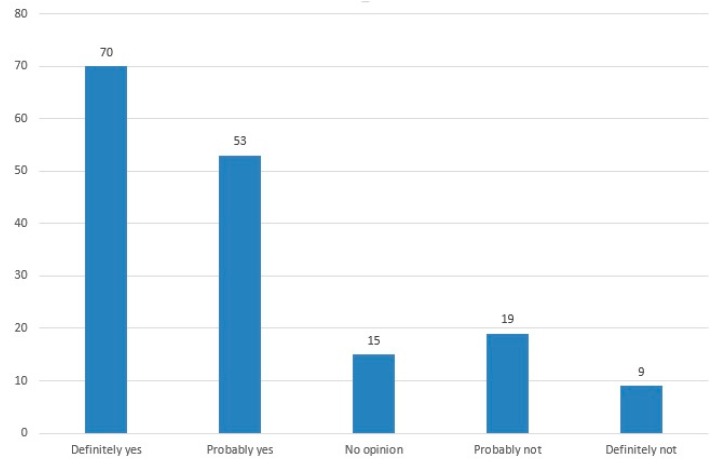
Future pregnancy plans.

Most pregnancies were wanted and planned (62.7%). Despite the planned nature of the pregnancy, the patients decided to terminate due to fetal disease. The unplanned pregnancies were also wanted. There was no statistical significance in this matter. A small number (3.3%) of the patients had sick children and because they were already familiar with the difficulties of looking after a sick child, they decided to terminate for fetal defects. Two out of the six mothers planned more pregnancies, the other four mothers were not planning any more pregnancies. No statistical significance was found as regards medical history, especially significant medical issues. Termination may have an impact on future reproductive plans. Eighteen percent of the patients were not planning more pregnancies (6% said “definitely not” and 12% said they would “probably not”) (Figure 1).

The majority (84.09%) of these women declared that this was due to the traumatic experiences related to their pregnancy.

The obtained statistical significance demonstrated that women who had been against pregnancy termination before undergoing termination, not only supported legal termination but also had more liberal views on abortion in a broader context, and without medical reasons (Table 2). Nearly one-fourth of the patients, before facing the dilemma of termination themselves, had been against termination for medical reasons (22.6%), of which 9.3% were definitely against terminations. Terminations were permissible in the view of 49.3%, with only 20% expressing definite permissibility (Table 2).

## 4. Discussion

The decision-making process about termination of pregnancy is a multi-stage process and involves many aspects of a woman’s life. Abortion may lead to significant psychological effects [13,14,15,16,17,18]. In a study between 1984–1997, which included 53,000 women who were informed about fetal defects at the prenatal stage, Schechtman et al. evaluated the decision-making process and demographic factors influencing the decision to terminate a pregnancy. They showed that most patients decided to terminate pregnancy in the case of central nervous system defects and genetic defects, or if the mother herself was older. Notably, the impact of the mother’s age suggests that older patients most likely have higher expectations with regard to pregnancy and are aware of a poorer quality of life in the case of giving birth to a child with severe defects [19]. In Great Britain, the average age of women who underwent pregnancy termination due to fetal defects was 33 years [8], which was higher than the age of the women who underwent termination and participated in the present study. Learman et al. [20] underlined that the average age of the patients examined most probably resulted from the place of the examination, i.e., the place where prenatal tests are performed and the age of women that are usually referred for such tests. In this study, the average age of the women was 33, although women over 35 constituted 45% of the respondents [20]. In another British study, the likelihood of abortion also seemed to grow with age, from 7% in the case of women aged 15–19 to 40% in women aged 30–34 [21]. Schechtman et al. suggested that younger patients may have been more sceptical about the information received and more often decided to continue with the pregnancy. The authors assumed that young mothers, when informed about treatment options for some defects, were not aware that despite the proposed treatment their child would remain severely disabled [19].

The decision to terminate a healthy pregnancy also seems to be influenced by the mother’s educational background [19]. Most likely, the physicians conducting the examination provide more precise and comprehensive information to women who were better educated and older. Conversely, less-educated women seemed to decide to terminate a pregnancy more frequently [18]. While examining the decision-making process in the case of fetal defects found in an ultrasound scan, with 43% of the defects found at the prenatal stage leading to termination, Bijma et al. showed that the decision to terminate a pregnancy was more often made at an earlier stage of the pregnancy after a diagnosis of severe defects affecting the central nervous system and genetic defects, in the case of past obstetric problems and less-educated mothers [22].

Our study indicated that the age and education of the mother does not have an impact on the decision; however, there was not much variation in the respondents’ educational background. The majority of the patients were educated, relatively young women. Similarly, in a study conducted in the US, there was no correlation between demographic characteristics and the propensity to terminate the pregnancy [23]. In another study assessing the social tendency of 2641 French women on deciding to terminate pregnancy, Combes et al. showed a correlation with income and family situation [5].

Women who had other children were more willing to undergo abortion than women who had not given birth [21]. A study assessing the risk of subsequent abortions confirmed that the risk grows in the case of older women and women who already have children [24]. Moreover, the risk reaches its peak in the case of older multigravidas. Interestingly, Lydon’s study suggested that women who had already had an abortion were less likely to undergo another abortion (28%) than women who had not undergone an abortion (69%) [24].

Our study does not confirm the hypothesis that women who have experienced a miscarriage or who have other children are more convinced about the termination of pregnancy. For example, a study conducted by Soderberg et al. demonstrated that areas of the city with higher abortion rates were characterized by lower income, a lower level of education, a higher percentage of foreigners and individuals receiving social welfare benefits [25]. Similarly, another study conducted in East and West Germany demonstrated differences in the approach to the topic of abortion depending on religious beliefs, which we did not notice in the present study [26]. Indeed, in this study, the vast majority of women declared they were Catholic (94.7%).

We did not find any studies regarding the future reproductive plans of women who were pregnant. In the present study, 18% of women did not plan any more pregnancies and the majority (84.09%) of these participants declared that this was due to the traumatic experiences related to their pregnancy. Nevertheless, it should be noted that the majority of patients did plan more pregnancies, which suggests their commitment and the decision to terminate a pregnancy was only based on fetal defects and not on social reasons.

An important aspect of the study was that most terminated pregnancies were planned, and if unplanned, then welcome. Only one patient was not satisfied with the occurrence of their pregnancy. Furthermore, and importantly, the majority of the women were highly aware of the need for medical prophylaxis (they presented for almost all recommended visits during their pregnancy and underwent preventive examinations at the recommended intervals). The majority of the respondents (86.7%) indicated that they visited a gynecologist for checkups at least once a year, which indicates the social awareness of diseases, concern for their own health and a satisfactory level of medical education.

A strength of our study is associated with the high number of participants compared to the number of procedures performed in the country. In the literature, we did not find records of studies carried out in other such culturally homogeneous environments, where abortion is still a taboo subject and a very emotional social issue.

The most important conclusion from the study is that views on the acceptability of termination of pregnancy and abortion may change with personal experience of this problem. Fewer than 50% of women accepted the abortion law on medical grounds, and nearly 23% were against it. However, despite such views they decided to terminate a pregnancy.

Several limitations should be taken into account with regard to the data interpretation in this study. More specifically, the study was conducted at a single facility situated in the capital of the country, which is why the majority of the patients had higher education degrees and lived in cities. As termination is not commonly available, the study group may not be representative of the rest of Poland. However, it shows the voice and worldview of the young generation, which differs from the dominant Catholic doctrine.

## 5. Conclusions

To conclude, our findings demonstrate that the decision to terminate pregnancy is an autonomous one, made by fully aware, well-educated women, who are mature in the emotional and biological sense, with stable personal circumstances. In addition, we found that external factors such as the environment, educational background, marital status, political and religious views do not affect the decision to terminate a pregnancy for medical reasons. Finally, the personal experience of pregnancy termination changes women’s views on the grounds for and availability of termination, abortion and future reproductive plans.

## Figures and Tables

**Table 1:** Sample characteristics [12].

**Age**	**Class**	**n**	**%**
	<20	2	1.33
	20–25	19	12.66
	25–30	44	29.33
	30–35	42	28.00
	35–40	29	19.33
	>40	4	9.33
**Place of living**	**Class**	**n**	**%**
	Provincial capital	44	29.33
	County town	56	37.33
	Smaller town	43	28.66
	Village	7	4.66
**Education**	**Class**	**n**	**%**
	Master’s degree	9	6.00
	Bachelor’s degree	79	52.66
	Secondary	59	39.33
	Primary	3	2.00
**Work category**	**Class**	**n**	**%**
	Medicine	18	12.00
	Administration	3	2.00
	Trade	4	2.66
	Banking	23	15.33
	Education	23	15.33
	Law	12	8.00
	Industry	13	8.66
	Services	12	8.00
	Construction	5	3.33
	Hotels and Gastronomy	7	4.66
	Agriculture	3	2.00
	Beauty	7	4.66
	Other	10	6.66
**Marital status**	**Class**	**n**	**%**
	Married	119	79.33
	Divorced	5	3.33
	Single	26	17.33
**Financial status**	**Class**	**n**	**%**
	Average	108	72.00
	Frequent Lack Of Money	1	0.67
	Occasional Lack Of Money	15	10.00
	Good	24	16.00
	Wealthy	1	0.67

**Table 2:** Past views on termination among patients who terminated pregnancy for medical reasons.

Before the Pregnancy, Were You Against Termination?	No.	%
Definitely not	30	20.0
Probably not	44	29.3
No opinion	41	27.3
Probably yes	20	13.3
Definitely yes	14	9.3
None	1	0.7
